# The Rationale and Evidence for Use of Inhaled Antibiotics to Control *Pseudomonas aeruginosa* Infection in Non-cystic Fibrosis Bronchiectasis

**DOI:** 10.1089/jamp.2017.1415

**Published:** 2018-06-01

**Authors:** Rajiv Dhand

**Affiliations:** Department of Medicine, University of Tennessee Graduate School of Medicine, Knoxville, Tennessee.

**Keywords:** aerosols, antibiotics, bronchiectasis, cystic fibrosis, inhalation therapy, nebulizer

## Abstract

Non-cystic fibrosis bronchiectasis (NCFBE) is a chronic inflammatory lung disease characterized by irreversible dilation of the bronchi, symptoms of persistent cough and expectoration, and recurrent infective exacerbations. The prevalence of NCFBE is on the increase in the United States and Europe, but no licensed therapies are currently available for its treatment. Although there are many similarities between NCFBE and cystic fibrosis (CF) in terms of respiratory symptoms, airway microbiology, and disease progression, there are key differences, for example, in response to treatment, suggesting differences in pathogenesis. This review discusses possible reasons underlying differences in response to inhaled antibiotics in people with CF and NCFBE. *Pseudomonas aeruginosa* infections are associated with the most severe forms of bronchiectasis. Suboptimal levels of antibiotics in the lung increase the mutation frequency of *P. aeruginosa* and lead to the development of mucoid strains characterized by formation of a protective polysaccharide biofilm. Mucoid strains of *P. aeruginosa* are associated with a chronic infection stage, requiring long-term antibiotic therapy. Inhaled antibiotics provide targeted delivery to the lung with minimal systemic toxicity and adverse events compared with oral/intravenous routes of administration, and they could be alternative treatment options to help address some of the treatment challenges in the management of severe cases of NCFBE. This review provides an overview of completed and ongoing trials that evaluated inhaled antibiotic therapy for NCFBE. Recently, several investigators conducted phase 3 randomized controlled trials with inhaled aztreonam and ciprofloxacin in patients with NCFBE. While the aztreonam trial results were not associated with significant clinical benefit in NCFBE, initial results reported from the inhaled ciprofloxacin (dry powder for inhalation and liposome-encapsulated/dual-release formulations) trials hold promise. A more targeted approach could identify specific populations of NCFBE patients who benefit from inhaled antibiotics.

## Introduction

Non-cystic fibrosis bronchiectasis (NCFBE) is a chronic inflammatory lung disease characterized by irreversible dilation of the bronchi, symptoms of persistent cough and expectoration, and recurrent infective exacerbations. The incidence of NCFBE in the United States is estimated at 52 cases per 100,000 population.^(1)^ Its prevalence is on the increase in the United States and Europe despite childhood vaccination programs and the widespread use of antibiotics to treat respiratory infections.^(2–4)^ According to an analysis of the Medicare Part B database, the prevalence of bronchiectasis increased by 8.74% each year between 2000 and 2007.^(5)^ This increase could be either due to a greater utilization of chest computed tomography (CT) scans resulting in an increase in diagnosis or due to unidentified etiologic factor(s) causing a real increase in prevalence in an aging population.^(5)^

**FIG. 1.**
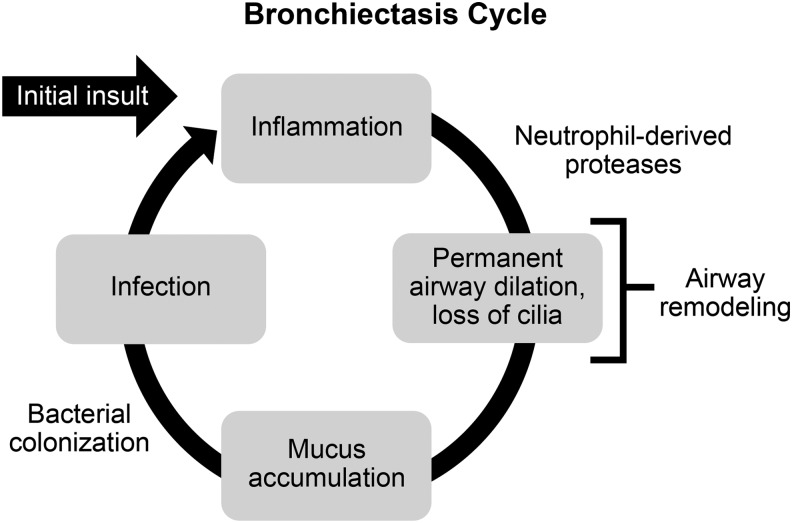
The “vicious circle” of bronchiectasis originally described by Cole.^(6)^

Progressive worsening of NCFBE is characterized by repeated cycles of bronchial bacterial infections that elicit a robust innate immune response, which leads to further airway damage and progressive loss of lung function (see “vicious circle” hypothesis developed by Cole^(6)^ in Fig. 1). Loss of lung function in severe cases of NCFBE leads to frequent hospitalizations, reduced quality of life, and increased mortality rates.^(7,8)^ Until recently, NCFBE received little attention because there are no approved drugs for its treatment. However, a recent revival of interest in this orphan disease has led to bronchiectasis registries being established in Europe^(9)^ and in the United States.^(10)^

NCFBE most commonly presents with symptoms of chronic cough, sputum production, fatigue, and hemoptysis (“wet” or “productive bronchiectasis”); however, some patients exhibit minimal or no symptoms (“dry” bronchiectasis).^(11,12)^ The reasons for a lack of symptoms in some patients with bronchiectasis could be due to the presence of localized or minimal disease and infrequent exacerbations due to adequate mucus drainage from the involved areas. The diagnosis of bronchiectasis is based on high-resolution CT scans of the chest showing that the internal diameter of the bronchus is larger than that of its accompanying vessel, or the bronchus fails to taper in the periphery of the chest.^(13)^ Airway wall thickening is often present, but this radiographic finding is not diagnostic of bronchiectasis.

Frequent infections and exacerbations characterize the course of illness in NCFBE, with ∼40% of patients experiencing ≥2 exacerbations annually.^(14)^ Exacerbations of NCFBE often require hospitalization and contribute to increased mortality.^(1)^ In patients with NCFBE, mortality ranges from 10% to 16% over an approximate 4-year observation period,^(15)^ and almost 30% over a 13-year follow-up period,^(16)^ due primarily to bronchiectasis or related respiratory failure. Poor lung function and advanced dyspnea scores correlate with a higher mortality. Compared with other causes of NCFBE, patients with idiopathic bronchiectasis have a lower death rate.^(17)^

## NCFBE and Cystic Fibrosis—Similarities and Differences

Although there are many similarities between NCFBE and the genetic disorder cystic fibrosis (CF) in terms of respiratory symptoms, airway microbiology, and disease progression, there are key differences, for example, in response to treatment,^(18)^ suggesting differences in pathogenesis (Table 1). CF develops at a young age and is associated with genetic mutation in the CF transmembrane conductance regulator gene, whereas the etiology in patients with NCFBE is heterogeneous and the cause cannot be established in many patients (idiopathic bronchiectasis).^(19)^ NCFBE is more prevalent than CF, its prevalence increases with advanced age, it is more frequent in women and the Asian population, and in contrast to CF occurs commonly in the lower lobe of the lungs, which makes the mucociliary clearance more difficult.^(20)^

**Table 1.** Differences Between Cystic Fibrosis and Non-cystic Fibrosis Bronchiectasis

	*Cystic fibrosis*	*Non-cystic fibrosis bronchiectasis*
Age	Young age	More common in older age
Sex	No gender difference in occurrence	More common in elderly women
Distribution	More common in upper lobes	More common in lower lobes
Etiology	Genetic mutation in CFTR gene complicated by infection	Generally postinfectious
Prevalence	Uncommon	Three to four times higher prevalence than CF; prevalence increases with age
Diagnosis	Sweat chloride level >60 mEq/L is diagnostic	Measurement of sweat chloride is not helpful for diagnosis
Comorbidities	Pancreatic insufficiency, sinusitis, airway hyper-responsiveness	Cardiovascular disease, COPD
Microbiology	*Pseudomonas*, *Acinetobacter*, *Burkholderia*	*Pseudomonas*, *Haemophilus*, *Moraxella*

CF, cystic fibrosis; CFTR, cystic fibrosis transmembrane conductance regulator; COPD, chronic obstructive pulmonary disease.

NCFBE is often underdiagnosed because it may be associated with other comorbidities, particularly chronic obstructive pulmonary disease. There are also noteworthy differences in therapeutic responses to various interventions: inhaled antibiotics have the potential to clear or “eradicate” initial *Pseudomonas aeruginosa* (*P. aeruginosa*) infection, postpone the development of chronic infection, reduce the frequency of exacerbations, and improve lung function in patients with CF, but similar clinical success has not been achieved in patients with NCFBE.^(7,21)^

**FIG. 2.**
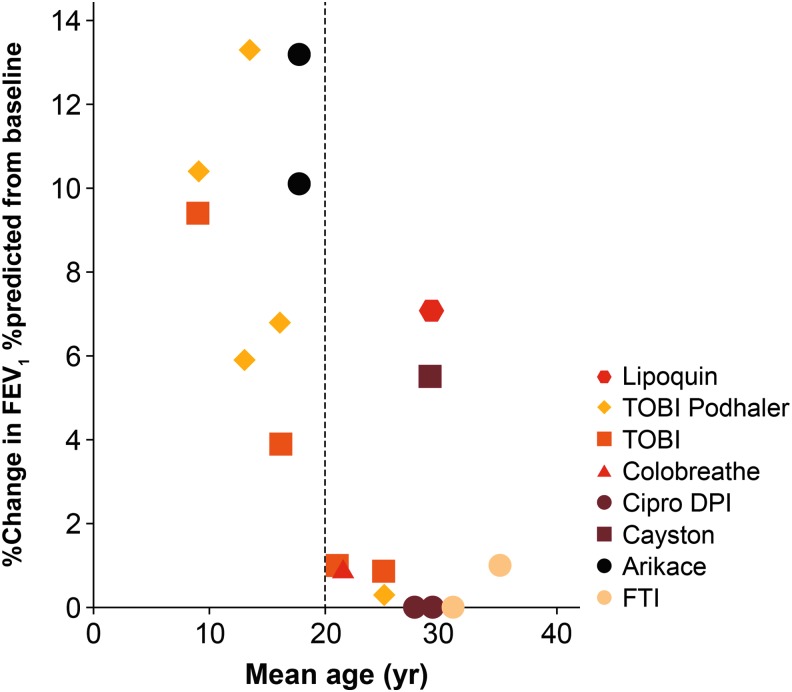
Improvements in lung function with inhaled antibiotics in patients with cystic fibrosis in relation to age. Reprinted from *Adv Drug Deliv Rev* 85, Cipolla et al., Comment on: Inhaled antimicrobial therapy—Barriers to effective treatment, e6–7, © 2015, with permission from Elsevier.^143^ DPI, dry powder for inhalation; FEV1, forced expiratory volume in 1 second; FTI, fosfomycin/tobramycin formulation; TOBI, tobramycin solution for inhalation.

In young patients with CF, “early” chronic infection with *P. aeruginosa* could be successfully cleared by antibiotic treatment with favorable outcomes.^(22)^ However, chronic infection with *P. aeruginosa* in patients with NCFBE is much more difficult to eradicate with antibiotics.^(23)^ The reasons for disparate responses to inhaled therapies in CF bronchiectasis and NCFBE are not well understood, but may depend on differences in the age of patients with CF and NCFBE. Improvement in lung function with inhaled antibiotics in patients with CF decreases with patients' age (Fig. 2).^(24,25)^ More advanced age of patients with NCFBE compared with the age of patients with CF who were included in clinical trials could explain the lack of improvement of lung function with inhaled antibiotics.

In patients with CF, irreversible changes in the airways related to persistent inflammation over many decades, remodeling and fibrosis, and mucus plugs, as well as biofilm formation could result in poorly ventilated areas that impair delivery of antibiotics to airways that are heavily colonized with bacteria.^(26)^ Moreover, antibiotics such as aminoglycosides and ciprofloxacin are less efficacious in anaerobic conditions that commonly exist in areas of poor ventilation, infection, and excessive mucus accumulation, whereas levofloxacin maintains its efficacy under such conditions.^(27,28)^ The occurrence of chronic inflammation and airway wall damage in patients with NCFBE produces a similar milieu to that described above^(29)^ and the efficacy of some inhaled antibiotics is likely to be impaired in such an environment.

The contribution of the bronchial circulation to differences in responses between CF and NCFBE also needs further evaluation. The bronchial arteries show enlargement and tortuosity in bronchiectasis with increased bronchopulmonary anastomosis.^(30,31)^ In patients with bronchiectasis, the principal source of blood supply is from the bronchial circulation; airway inflammation and remodeling contribute to an increase in the airway mucosal vasculature accompanied by new vessel growth from pre-existing vessels and dilatation of existing vessels.^(32,33)^ Moreover, infection with *P. aeruginosa* induces vascular endothelial growth factor *in vitro* and *in vivo.*^(34)^ The fenestrations in the endothelium of the bronchial capillaries,^(35)^ especially in the newly formed vessels, facilitate drug penetration, and the presence of inflammation further enhances the permeability of the vessels.^(36)^ The close proximity of the submucosal bronchial venous system to the airway lumen allows for rapid absorption of inhaled drugs.^(37)^ Thus, the bronchial circulation could play a role in more rapid absorption of antibiotics, and rapid clearance of antibiotics after deposition could limit the duration of their effect at the site of infection unless formulations have adequate residual lung time. However, evaluation of bronchoalveolar lavage fluid found high levels of inflammatory markers in both patients with CF and NCFBE.^(38)^ The levels of neutrophil elastase (and neutrophils), matrix metalloprotease (MMP)-2, and MMP-9 in patients with NCFBE were higher than those in healthy controls but were lower compared to patients with CF.^(38)^ Notably, there are no reported differences in the bronchial circulation or bronchial blood flow to damaged airways that could explain the disparate response to inhaled therapies in patients with NCFBE compared with those with CF.^(33)^

In bronchiectasis, there is hypertrophy of the mucus-secreting glands, hyperplasia and metaplasia of goblet cells, and an increase in mucus production.^(39)^ Disturbances in mucociliary clearance are central to the development of bronchiectasis and lead to mucus accumulation (Fig. 1) and plugging of small and large airways with mucus as seen on high-resolution chest CT scans. The nature of the mucus is also altered by the presence of chronic inflammation, and properties of the mucus in children with NCFBE differ from those observed in other respiratory diseases.^(40)^ Airway clearance techniques are safe in patients with stable bronchiectasis, although their role in patients with acute exacerbations is not yet clearly established.^(41)^

In patients with CF, DNA and F-actin filaments released from apoptosis of neutrophils and from damaged epithelial cells are mainly responsible for the increase in viscosity of sputum, and rhDNase reduces the viscosity of sputum by depolymerizing DNA filaments.^(42)^ In contrast, there is a paucity of DNA polymers in sputum of patients with NCFBE and clearance by cough is greater than sputum in CF patients. Moreover, inhalation of rhDNase may not be as effective in reducing sputum viscosity in adults with NCFBE.^(43,44)^

Alterations in the properties of mucus in patients with bronchiectasis may impair response to antibiotic therapy. The long and branching mucin glycoproteins form a mesh that impedes the transport of aminoglycosides, beta-lactam, and fluoroquinolone antibiotics *in vitro*,^(45)^ especially for liposomal formulations of aminoglycosides.^(46)^ Moreover, binding to certain large molecules in sputum could reduce antibiotic efficacy. Aminoglycosides bind to mucin, especially in the presence of free DNA, and in an acidic environment.^(47–50)^ Mucus binding has not been observed with beta-lactam antibiotics^(51)^ and further studies are needed to determine if similar interactions occur between mucus and fluoroquinolones or macrolide antibiotics.

Changes in the mucus and antibiotic binding may impede the penetration of antibiotics to the surface of epithelial cells. While high concentrations of intraluminal antibiotics achieved after inhalation are effective at clearing organisms in the sputum, they may not be effective in penetrating through the mucus barrier into the tissues, and suboptimal antibiotic concentrations at the site of infection could impair their ability to eradicate infection in the airways and lung parenchyma.

The response to mucoactive agents has been variable in patients with NCFBE. Their efficacy in addition to long-term antibiotics needs to be explored in future studies, particularly in view of the *in vitro* observation that prior deposition of mannitol delayed the transport of ciprofloxacin hydrochloride in a Calu-3 air–interface cell model.^(52)^ The delay in ciprofloxacin hydrochloride transport across the epithelium could provide dual benefits by reducing the dosing frequency and by maintaining higher antibiotic concentrations at the site of infection for a longer period.

## Role of *Pseudomonas aeruginosa* in NCFBE

Frequently identified bacterial pathogens in sputum isolates from patients with NCFBE include *P. aeruginosa*, *Moraxella catarrhalis*, and *Haemophilus influenzae*.^(53)^ *P. aeruginosa* infections are reported in as many as one-fourth to one-half of patients and are associated with the most severe forms of bronchiectasis (defined as requiring hospital admission or characterized by the development of resistance to oral antibiotics), which are linked to higher morbidity and mortality.^(7,8)^ Nontuberculous mycobacteria and gram-positive organisms (*Streptococcus pneumoniae* and *Staphylococcus aureus*) are also found.^(54–57)^ Sputum studies with molecular techniques, instead of bacterial cultures, have identified conventional pathogens such as *P. aeruginosa* and *H. influenzae* as well as anaerobic organisms such as Prevotella and Veillonella organisms whose pathogenicity is not clearly established.^(58)^

Isolation of *P. aeruginosa* has been identified as an independent predictor of accelerated decline of lung function in patients with NCFBE.^(59)^ Furthermore, patients with *P. aeruginosa* infections score higher than infections with other bacterial pathogens on the Bronchiectasis Severity Index (BSI), which accurately predicts lung function decline, mortality, hospital admissions, exacerbations, quality of life, and respiratory symptoms in patients with bronchiectasis.^(60,61)^ *P. aeruginosa* is also a key determinant in another severity scoring system, the FACED score.^(62)^ The BSI and FACED scores are multidimensional tools that are predictive of mortality for up to 15 years after diagnosis of NCFBE.^(63)^

Key strategies for the management of *P. aeruginosa* in patients with NCFBE cover three main stages: (1) eradication of the pathogen on first isolation; (2) treatment during acute exacerbations; and (3) management of chronic infections.^(8)^ Data on the eradication of *P. aeruginosa* on first isolation are currently limited; however, a small, retrospective study investigating 30 patients with NCFBE found that eradication of the pathogen was initially successful in 80% of patients with a combination of intravenous, oral, or inhaled antibiotics, resulting in a reduced exacerbation rate following eradication.^(22)^ Nevertheless, 46% of these patients were positive again for *P. aeruginosa* after a median follow-up of 6.2 months.^(22)^

Chronic infections with *P. aeruginosa* require long-term antibiotic therapy to reduce the bacterial load and therefore the frequency and severity of exacerbations.^(8)^ The frequency of their acute exacerbations may determine whether a patient is considered a candidate for long-term antibiotic therapy. Three acute exacerbations per year were considered to be an appropriate threshold for therapy by the British Thoracic Society^(64)^ and by 46% of respondents in a live electronic polling session that was conducted at an Expert Forum of the European Respiratory Society in 2014.^(8)^

The simplest method of administering long-term antibiotics is by the oral route. Commonly used oral macrolide antibiotics have immunomodulatory properties in addition to their antibacterial effects. Recently, three randomized, double-blind, placebo-controlled studies in patients with NCFBE reported that azithromycin or erythromycin administered orally for 6–12 months was generally well tolerated and led to a reduction in exacerbation rate, and a reduced rate of lung function decline (Table 2).^(65–67)^

**Table 2.** Randomized, Double-Blind, Placebo-Controlled Trials of Oral Macrolides in Non-cystic Fibrosis Bronchiectasis

	*EMBRACE^65^*		*BLESS^66^*		*BAT^67^*	
	*Placebo*	*Azithromycin*	*Placebo*	*Erythromycin*	*Placebo*	*Azithromycin*
Patient, *n*	70	71	58	59	40	43
Male/female, *n*	20/50	23/48	25/33	21/38	12/28	18/25
Age, years	59.0	60.9	63.5	61.1	64.6	59.9
*Study duration*	*6 months*		*12 months*		*12 months*	
FEV_1_% predicted at baseline	67.3	67.1	70.1	66.9	82.7	77.7
Change in FEV_1_ from baseline, L	−0.04	0	−4.0	−1.6	−0.10^a^	1.03^a^
SGRQ at baseline, total score	36.6	31.9	38.1	36.7	40.2	40.6
Change in SGRQ total score from baseline	−1.92	−5.17	−1.3	−3.9	−4.12	−12.18
Exacerbation rate in 12 months before trial	3.93	3.34	NR^b^	NR^b^	4.0	5.0
Total no. of exacerbations over 12 months	178^c^	109^c^	114	76	78	39
Annual exacerbation rate, patient/year	2.73^c^	1.58^c^	1.97	1.27	1.95	0.91
Patients with ≥1 exacerbation, *n* (%)	58 (82.9)	44 (62.0)	42 (72.4)	39 (66.1)	32 (80.0)	20 (46.5)
NNT to prevent one patient experiencing an exacerbation over 12 months^d^	5		16		3	

Data are means unless otherwise stated.

Reprinted from *Respiratory Medicine*, 108(10), Haworth CS, Bilton D, Elborn JS, Long-term macrolide maintenance therapy in non-CF bronchiectasis: Evidence and questions, 1397–1408, © 2014.^144^

^a^ Data change per visit (every 3 months), *F*_1,78.8_ = 4.085, *p* = 0.047.

^b^ BLESS study did not present exacerbation rate, but did present the number of patients with five or more exacerbations in the year preceding the trial (*n* = 20 and 22 for placebo and erythromycin, respectively).

^c^ EMBRACE was a 6-month study but presented annualized data for exacerbations.

^d^ Calculated as 1/absolute risk reduction (proportion with event [placebo] − proportion with event [intervention]). Values presented are the published NNT for BAT and estimates by the authors for EMBRACE and BLESS, based on the percentage of patients with exacerbation events.

BAT, Bronchiectasis and Long-term Azithromycin Treatment study; BLESS, Bronchiectasis and Low-dose Erythromycin study; EMBRACE, Effectiveness of Macrolides in patients with Bronchiectasis using Azithromycin to Control Exacerbations study; FEV_1_, forced expiratory volume in 1 second; NNT, number needed to treat; NR, not recorded; SGRQ, St. George's Respiratory Questionnaire.

Inhaled antibiotics in patients with NCFBE are primarily directed at gram-negative organisms, especially *P. aeruginosa*. Several classes of inhaled antibiotics, including aminoglycosides, cephalosporins, colistin, fluoroquinolones, and aztreonam, have been used in patients with NCFBE with the premise that airway and systemic inflammation are related to the bacterial load, and a decrease in the bacterial density in the airways could reduce airway inflammation and lung damage, leading to improved clinical outcomes.^(68,69)^ Most of these antibiotics have a concentration-dependent killing effect and the rationale is to “hit hard and hit fast” to maximize efficacy while reducing the chances of development of resistance^(54)^ and systemic toxicity. In patients with CF, the rationale for the 28-day on and 28-day off cycle of administration of inhaled antibiotics depends on the peak increase in lung function after 28 days of continuous antibiotic administration and the likelihood that the 28-day off period reduces the selective pressure for emergence of antibiotic-resistant organisms.^(3,70)^

Administration of intravenous antibiotics is an option for patients who are not responding to oral or inhaled therapy or if there is no suitable oral or inhaled alternative. Some centers use “pulsed intravenous antibiotics.” In this approach, regular intravenous antibiotics are self-administered at home or by the community health team for 7–14 days at set intervals of 6–8 weeks.^(71)^ However, despite small benefits, including a small reduction in mean total exacerbation frequency and a significant reduction in hospital bed days, this approach has not been widely adopted. In addition to the route of administration and bacteriology, optimal therapy also needs to consider patient preferences and comorbidities. It may be more difficult for patients to have a high level of compliance needed for effective antibiotic therapy with nebulizers compared with oral administration of antibiotics.^(72)^ The use of nebulized antibiotics requires close monitoring of adherence to treatment.

### Challenges in treating *Pseudomonas aeruginosa* in the lung

**FIG. 3.**
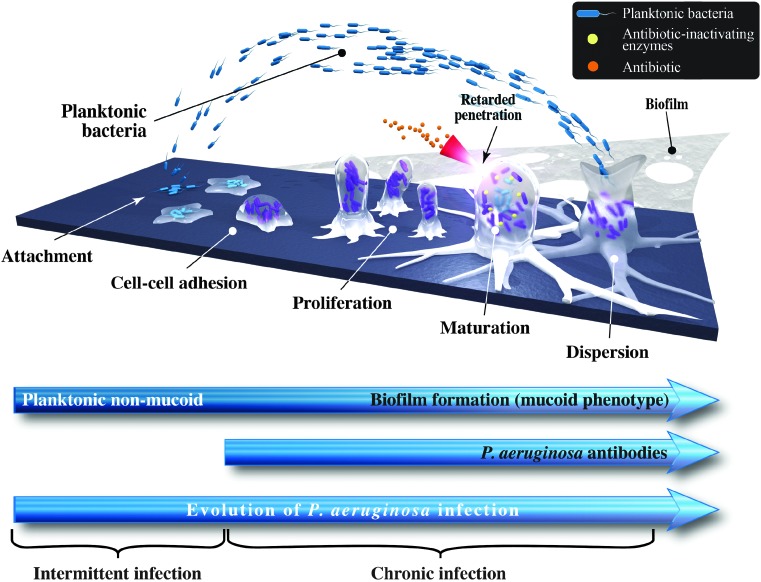
Evolution of *Pseudomonas aeruginosa* infection in the lung from intermittent to chronic infection.

Systemically delivered antibiotics do not penetrate optimally into lung tissue.^(73)^ In the initial stages, the nonmucoid phenotype of *P. aeruginosa* is present as planktonic organisms within sputum.^(74–76)^ These nonmucoid strains do not form biofilm or induce antibody responses and do not cause much lung damage, and they could be treated with inhaled antibiotics or a combination of oral and intravenous antibiotics.^(77)^ Suboptimal levels of antibiotics in the lung increase the mutation frequency of *P. aeruginosa* and lead to the formation of mucoid strains of *P. aeruginosa* that are characterized by a protective alginate, polysaccharide matrix known as a “biofilm.” These biofilms provide a physical and chemical barrier that allows *P. aeruginosa* to evade immune cells such as phagocytes and host antibodies, and makes it highly resistant to systemically administered antibiotics (Fig. 3).^(56,59,78–82)^

Within biofilms, bacteria coordinate their biological activity and synchronize gene expression via quorum sensing.^(83)^ In addition, the matrix of the biofilm prevents access of antibiotics to bacterial cells. In *in vitro* studies, alginate not only limits diffusion of aminoglycosides and beta-lactam antibiotics it also appears to bind aminoglycosides and polymyxin B.^(27,84–87)^ In a preclinical study, a liposomal formulation of amikacin was shown to have greater ability to penetrate *P. aeruginosa* biofilms compared with the free drug.^(88)^ Similarly, *in vitro* studies demonstrated that liposomal ciprofloxacin was able to penetrate biofilms from clinical isolates of *P. aeruginosa* with 99% reduction in cell viability at a concentration of 1 μg/mL.^(2)^ Another phenomenon that limits the efficacy of antibiotics within biofilms is the presence of “persister cells.” This small fraction of bacteria within the colony is in a dormant or nongrowing state and antibiotics that typically target actively dividing cells are unable to kill them. Persister cells that remain after antibiotics kill the susceptible organisms are able to reconstitute the biofilm after the treatment has concluded.^(89,90)^

Failure to eradicate *P. aeruginosa* using antibiotic therapy and development of mucoid strains is associated with subsequent transition to a chronic infection stage, requiring long-term antibiotic therapy to control the infection.^(61,62)^

Chronic or frequent subtherapeutic exposure to antibiotics (i.e., levels lower than the minimum inhibitory concentration [MIC]) further increases the ability of *P. aeruginosa* to develop antibiotic resistance. Results from a meta-analysis revealed that the risk of antibiotic resistance in patients with bronchiectasis increases more than threefold with long-term (≥4 weeks) antibiotic therapy.^(91)^ Furthermore, in a study of 89 patients with bronchiectasis, 10.1% of sputum isolates with *P. aeruginosa* showed antibiotic resistance after a mean 5.7 years of follow-up compared with 3.4% of sputum isolates at the initial assessment visit.^(92)^ Treatment with a broad-spectrum antibiotic also may have a negative effect on the diversity of the lung microbiome, which may result in reinfection with *P. aeruginosa*, even in the presence of concurrent antipseudomonal antibiotics.^(93)^

### Strategies to reduce development of resistance

Strategies to reduce the development of antibiotic resistance include alternating periods with antibiotic therapy with treatment-free periods; rotating use of different antipseudomonal antibiotics; using antibiotic combination therapy; and using antibiotic adjuvants such as gallium, antimicrobial peptides, and antibiofilm compounds, including alginate oligosaccharides that facilitate antibiotic penetration of the bacterial cell.^(81,94–96)^ One of these alginate oligosaccharides (OligoG CF-5/20) was found to be safe for inhalation both in healthy and in chronically diseased lung patients and is currently undergoing phase IIb trials in CF patients.^(97)^ Furthermore, a new family of compounds (“peptidomimetics”), mimicking antimicrobial peptides and showing activity against *P. aeruginosa*,^(96,98)^ are currently being evaluated in preclinical studies.^(95,96)^

Other strategies to reduce the emergence of bacterial resistance include use of efflux pump inhibitors, suppression of hydroxyl-free radical generation, and pharmacokinetic/pharmacodynamic approaches. By achieving high levels of drug exposure in the respiratory tract, inhaled antibiotics may have the potential to reduce the occurrence of antibiotic resistance. A meta-analysis derived from seven trials (*n* = 445) that investigated the emergence of bacterial resistance in a pooled population with stable NCFBE found no statistically significant difference between the emergence of bacterial resistance in patients receiving inhaled antibiotic therapy (amikacin, aztreonam, ciprofloxacin, gentamicin, colistin, or tobramycin) compared with patients receiving either placebo or symptomatic treatment with oxygen, bronchodilators, and corticosteroids (7.8% vs. 3.5%, respectively; risk ratio [RR] [95% confidence interval (CI)] 1.68 [0.62–4.52]; *p* = 0.31).^(99)^

**FIG. 4.**
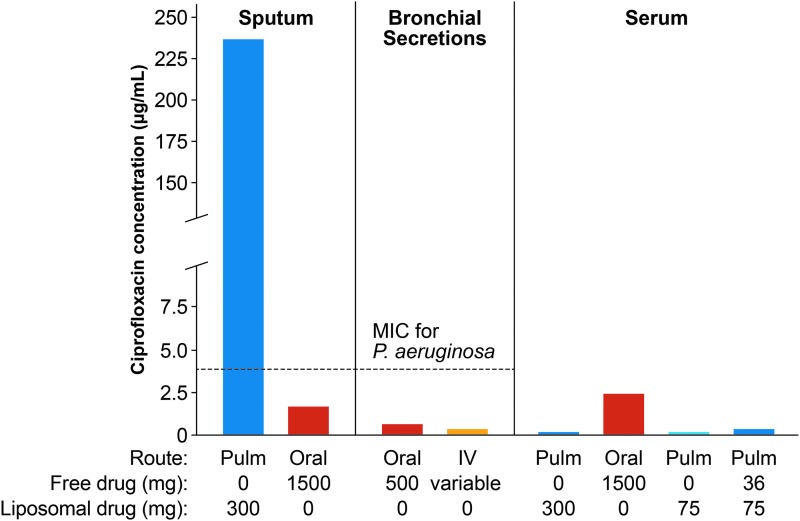
The value of inhaled delivery using the antibiotic ciprofloxacin as an example. Reproduced from Cipolla et al.^(2)^ IV, intravenous administration; MIC, minimum inhibitory concentration; Oral, oral administration; Pulm, inhaled administration.

Inhaled antibiotic formulations are associated with minimal systemic toxicity and adverse events compared with oral/intravenous routes of administration.^(18,54,88)^ This is illustrated in Figure 4, using the fluoroquinolone antibiotic ciprofloxacin as an example. Maximum oral doses of ciprofloxacin (750 mg twice a day, as recommended by the British Thoracic Society^(100)^) to patients with CF led to an antibiotic concentration in sputum that was lower than the MIC required for *P. aeruginosa* (Fig. 4). In contrast, inhaled ciprofloxacin resulted in drug concentrations in the sputum that were ≥50-fold greater than the MIC for *P. aeruginosa*.^(2)^ At the same time, serum drug concentrations with inhaled ciprofloxacin were considerably lower than those achieved using oral administration, minimizing the possibility of systemic toxicity and adverse events (Fig. 4). It is worth noting that lung concentrations of inhaled antibiotics may vary depending on the particle size, ability to reach the smaller airways, disease state, formulation used, and occurrence of side effects such as bronchoconstriction and cough. Concerns about emergence of resistance with long-term use of inhaled antibiotics because of lower concentrations achieved in the distal airways and lung parenchyma due to mucus plugging and airway obstruction were not substantiated in clinical studies.^(54,99)^

Initially, drug solutions designed for intravenous antibiotic administration were used for inhalation. Later, specialized suspension formulations and, finally, liposomal formulations, such as amikacin and ciprofloxacin, were developed. Formulations that are suitable for inhalation should be sterile, preservative free, and nonpyrogenic. They also should be adjusted for the lung environment with a suitable pH (range 4.0–8.0, but preferably closer to neutral pH), osmolarity (150–1200 mOsm/L), and tonicity.^(101,102)^ A specifically formulated solution for inhalation could minimize adverse effects, such as airway irritation, and increase delivery efficiency. More recently, dry powder formulations using PulmoSphere technology have the ability to carry a higher payload of antibiotics. The pulmonary deposition of the hollow porous particles within these powders is independent of the patient's inspiratory flow.^(103,104)^ The ability to inhale at low inspiratory flow rates without compromising drug deposition could reduce the incidence of cough and bronchospasm in patients.^(53,105)^

Treatment failures due to lack of adequate concentration of antibiotics, inadequate time of retention in the lungs, and compliance issues with more than once-daily dosing could be ameliorated by use of sustained-release formulations, such as liposomes and those with solubility limited dissolution rate using salts of polylactic-co-glycolic acid (PLGA). By using novel inhaled antibiotic formulations, such as dry powder for inhalation (DPI) ciprofloxacin, nebulized liposomal amikacin, or nebulized dual-release liposomal ciprofloxacin, investigators have observed good efficacy and safety in clinical trials.^(106)^ Clinical outcomes with inhalable PLGA formulations of tobramycin, clarithromycin, and ciprofloxacin have not been reported.^(107)^

In addition to the development of novel antibiotic formulations, rapid technological developments in portable aerosol delivery devices have contributed to the ability to achieve a higher efficiency of drug delivery to the lung.^(108,109)^ With the jet nebulizer, the efficiency of tobramycin delivery is only 5%–15%.^(110–112)^ The whole lung deposition of dry powder tobramycin with the PulmoSphere formulation was reported to be ∼34% in healthy volunteers^(110)^ and the delivery time was only 2–3 minutes. Tobramycin solution for inhalation (Novartis, New York, NY) is administered twice a day with specific jet nebulizers, with each dose requiring 10–15 minutes to deliver. Aztreonam lysine inhalation solution (Cayston, Gilead, Foster City, CA) requires three times a day administration over 2–3 minutes using the Altera vibrating mesh nebulizer system (PARI Respiratory Equipment, Midlothian, VA). These newer delivery systems are portable and efficient for inhaled drug delivery. Other antibiotics are being delivered with delivery systems that are not specifically approved for use with these formulations, or have not been approved for clinical use.

## Clinical Evidence Supporting Use of Inhaled Antibiotics

The majority of the clinical evidence on the use of inhaled antibiotics comes from studies investigating pseudomonal infections in CF and nosocomial pneumonia in ventilated patients. The greatest success of use of inhaled antibiotics has been for the treatment of pseudomonal infections in CF.^(3,4,113,114)^ For this indication, inhaled forms of tobramycin^(115)^ and aztreonam^(20)^ are approved in the United States and Europe, and colistimethate^(116)^ in Europe. In patients with CF, inhaled antibiotics slow the rate of decline of lung function, delay onset of chronic infection with *P. aeruginosa*, reduce the rate of exacerbations and hospitalizations, and improve quality of life.^(3,4,113,114)^ Some formulations that have been tested in patients with CF but not in those with NCFBE include levofloxacin nebulizer solution,^(117)^ DPI colistin,^(118)^ and fosfomycin/tobramycin nebulizer solution.^(119)^

Macrolides have positive immunomodulatory effects, including reduced airway inflammation and airway damage, with decrease in mucus hypersecretion and less biofilm formation.^(57,120)^ Long-term use of oral macrolides in patients with NCFBE improves respiratory symptoms and quality of life, while reducing the decline in lung function and the frequency of acute exacerbations (Table 2).^(121)^ Some inhaled macrolide formulations have not been clinically tested.^(122–126)^ Concerns with long-term use of macrolides include the potential for macrolide resistance and emergence of new pathogens.^(127)^ Increasing use of macrolides, especially azithromycin, is associated with a consistent increase in macrolide resistance at the population level.^(25)^ Increasing macrolide-resistant strains in the community could influence the clinical outcomes of macrolide therapy, especially when a large number of patients use macrolides on a long-term basis and become carriers of macrolide-resistant organisms.^(25)^

Long-term intermittent therapy with ciprofloxacin DPI could reduce the frequency of acute exacerbations in patients with NCFBE colonized with respiratory bacterial pathogens.^(53,128,129)^ Patients inhale ciprofloxacin inhalation powder from one capsule of ciprofloxacin DPI 32.5 mg twice daily using a pocket-sized, T-326 breath-actuated inhaler. The proposed regimen for ciprofloxacin DPI is to use cycles of 14 days on drug and 14 days off drug (or 28 days on/28 days off). The DPI facilitates lung deposition and achieves a high local concentration of ciprofloxacin in the lung with a longer half-life than ciprofloxacin hydrochloride.^(105)^ A recent scintigraphic study with the ciprofloxacin DPI reported mean lung deposition of 53% in patients with NCFBE, 51% in patients with chronic obstructive pulmonary disease, and 51% to 53% in healthy volunteers. After inhalation of ciprofloxacin, there were no episodes of bronchospasm or clinically significant changes in lung function, and systemic exposure to ciprofloxacin was low.^(130)^

As previously mentioned, although pathogens are similar in CF and NCFBE, the rate of progression and extent of changes in the lung are not the same, suggesting that there are differences in pathogenesis (Table 1). Therefore, outcomes of inhaled antibiotic therapy should not be assumed to be the same in CF and NCFBE.^(18)^

### Clinical trials evaluating the use of inhaled antibiotic therapy in NCFBE

There are currently no drugs that are specifically approved for treatment of patients with NCFBE. In NCFBE patients with *P. aeruginosa* infections, the British Thoracic Society recommends monotherapy with oral ciprofloxacin (500–750 mg twice a day) as first-line treatment.^(100)^ However, the British Thoracic Society guidelines do not currently make any recommendations with regard to the use of inhaled ciprofloxacin as monotherapy for NCFBE,^(100)^ until further evidence is available.

Table 3 provides an overview of completed and ongoing clinical trials that have evaluated inhaled antibiotic therapy for NCFBE. The efficacy and tolerability profiles of some commercially available inhaled antibiotics in patients with NCFBE do not appear to be as promising as those in patients with CF. Inhaled tobramycin resulted in a statistically significant reduction in *P. aeruginosa* bacterial load; however, a high proportion of patients (50%) had respiratory adverse events.^(95)^ A randomized, placebo-controlled trial of colistin in 144 patients did not meet the primary endpoint of time to exacerbation.^(72)^ Two large, randomized, double-blind, phase 3 trials that evaluated aztreonam lysinate in a total of 540 patients reported no difference versus placebo across outcome measures.^(20)^ There was no significant difference in quality-of-life scores, and treatment-related adverse events were more common in the aztreonam-treated group compared with the placebo group.^(19)^

**Table 3.** Overview of Clinical Trials Investigating Inhaled Antibiotic Therapy for Non-cystic Fibrosis Bronchiectasis

*Study*	*Study design*	*No. of randomized patients*	*Intervention*	*Key outcome measures*	*Efficacy results*	*Safety results*
Barker et al.^(20)^	Two randomized, placebo-controlled, phase 3 trials	AIR-BX1: *n* = 266	Aztreonam for inhalation	Change in QOL-B respiratory symptom scores to week 4	No difference versus placebo across outcome measures	Treatment-related AEs were more common in the aztreonam group versus placebo
		AIR-BX2: *n* = 274				
Drobnic et al.^(141)^	Randomized, placebo-controlled, two-period, crossover trial	*n* = 30	Inhaled tobramycin BID in two cycles, each for 6 months	Number of exacerbations, number of hospital admissions and number of hospital admission days	No difference in number of exacerbations (*p* = 0.330); significant reduction in hospital admissions (*p* = 0.038) and days in hospital (*p* = 0.047) in treatment group	Inhaled tobramycin was associated with bronchospasm in 10% of patients
Wilson et al.^(53)^ (Clinicaltrials.gov identifier: NCT00930982)	Randomized, placebo-controlled, phase 2 trial	*n* = 124	Ciprofloxacin DPI 32.5 mg or placebo BID 28 days on/28 days off	Effect on total bacterial density of predefined pathogens in sputum on day 28	Ciprofloxacin DPI resulted in a statistically significant reduction in total bacterial load on day 28 (*p* < 0.001)	No significant differences between the ciprofloxacin DPI and placebo arms; the incidence of bronchospasm was low
RESPIRE-1^(132)^ (Clinicaltrials.gov identifier: NCT01764841)	Randomized (2:1), placebo-controlled, phase 3 trial	*n* = 416	Ciprofloxacin DPI 32.5 mg or placebo BID 28 days on/28 days off or 14 days on/14 days off over 48 weeks	Time to first pulmonary exacerbation versus pooled placebo and frequency of exacerbation versus matched placebo	Ciprofloxacin DPI significantly reduced number of exacerbations (*p* = 0.0005) and exacerbation frequency (*p* = 0.0061) versus placebo with the 14-day on/14-day off regimen. No significant changes versus placebo in exacerbations or exacerbation frequency were observed with the 28-day on/28-day off regimen	No difference in serious treatment-emergent AEs with the 14-day regimen (16.9%), the 28-day regimen (19.9%), or placebo (23.4%). Rates of discontinuation because of respiratory AEs were similar
RESPIRE-2^(133)^ (Clinicaltrials.gov identifier: NCT02106832)	Randomized (2:1), placebo-controlled, phase 3 trial	*n* = 521	Ciprofloxacin DPI 32.5 mg BID 28 days on/28 days off or 14 days on/14 days off over 48 weeks	Time to first pulmonary exacerbation versus pooled placebo and frequency of exacerbation versus matched placebo	Ciprofloxacin DPI did not significantly prolong time to first exacerbation or reduce exacerbation frequency to predefined significance thresholds	Ciprofloxacin DPI was well tolerated in both regimens
ORBIT-1^(2,59)^ (Clinicaltrials.gov identifier: NCT00889967)	Randomized, placebo-controlled, double-blind trial	*n* = 96	Ciprofloxacin for inhalation (150 or 100 mg once daily) for one cycle of 28 days on and 28 days off	Mean change in *Pseudomonas aeruginosa* density in sputum (log_10_) CFU/g of sputum from baseline to day 28	Significant mean decreases from baseline in *P. aeruginosa* CFU at day 28 after 150 mg dose of 3.5 log_10_ (*p* < 0.001) and after 100 mg dose of 4.0 log_10_ units (*p* < 0.001)	Treatment was well tolerated, with no statistically significant differences between active treatment and placebo groups in the number of patients experiencing ≥1 respiratory treatment-emergent event
Serisier et al.^(131)^ ORBIT-2	Randomized, placebo-controlled, phase 2 trial	*n* = 42	Dual-release liposomal ciprofloxacin for inhalation (150 mg) and free ciprofloxacin (60 mg) versus placebo 28 days on/28 days off in three cycles	Mean change in sputum *P. aeruginosa* density from baseline to day 28 (first treatment cycle)	At day 28, dual-release ciprofloxacin resulted in a reduction from baseline of mean (SD) −4.2 (3.7) log_10_ CFU/g in sputum *P. aeruginosa* density versus a change from baseline of −0.08 (3.8) log_10_ CFU/g in the placebo group (*p* = 0.002)	Incidence of systemic AEs similar between two arms; there were fewer pulmonary AEs in the ciprofloxacin arm versus Placebo
ORBIT-3/ORBIT-4^(134–136)^ (Clinicaltrials.gov identifier: NCT01515007, NCT02104245)	Randomized (2:1), placebo-controlled, phase 3 trials	Five hundred eighty-two patients were enrolled (ORBIT-3, *n* = 278; ORBIT-4, *n* = 304)	Dual-release ciprofloxacin for inhalation (ARD-3150; liposome-encapsulated ciprofloxacin [150 mg/3 mL] and free ciprofloxacin [60 mg/3 mL]) for 28 days on/28 days off for six cycles	Time to first pulmonary exacerbation, frequency of all and severe pulmonary exacerbations	ARD-3150 was associated with an increased median time to first exacerbation >2 months versus placebo; the result was significant for ORBIT-4, but not for ORBIT-3. Significant reductions in frequency of all and severe exacerbations were observed for ARD-3150 versus placebo in ORBIT-4, but not ORBIT-3. In the pooled analysis of the two trials, ARD-3150 led to a significant increase versus placebo in median time to first exacerbation that required antibiotics and a significant reduction in PE frequency; it also significantly reduced PA sputum density during each on-treatment period	Rates of TEAEs and serious TEAEs were similar in both treatment groups
Murray et al.^(88)^	Randomized, controlled trial	Sixty-five patients with NCFBE and chronically infected sputum	Nebulized gentamicin 80 mg BID or placebo (0.9% saline) BID for 12 months	Sputum bacterial density reduction of at least 1 log unit, sputum purulence, exacerbation rate, time to first exacerbation	Baseline sputum bacterial density was 8.02 log_10_ CFU/g. Gentamicin treatment significantly reduced sputum bacterial density to 2.96 log_10_ CFU/g versus 7.67 log_10_ CFU/g in the placebo group (*p* < 0.0001)	Gentamicin was well tolerated, 7 (22%) patients reported bronchospasm but 5 continued treatment with albuterol use as a bronchodilator. Only two patients withdrew owing to poor tolerability, the same as in the placebo arm
					Gentamicin treatment reduced purulence, exacerbation rates, and increased time to first exacerbation. However, 3 months post-treatment, there was no difference between treatment and placebo groups in any of these parameters	
Barker et al.^(142)^	Randomized, placebo controlled trial	Randomized 74 patients with chronic *P. aeruginosa* colonization	Nebulized tobramycin 300 mg BID or placebo (quinine/saline) BID for 28 days	*P. aeruginosa* density in sputum, medical condition, and emergence of resistance	Tobramycin significantly reduced *P. aeruginosa* density by 4.54 log_10_ CFU/g sputum compared with a mean increase of 0.02 log_10_ CFU/g sputum in placebo patients (*p* < 0.01) Tobramycin improved medical condition at week 6. No significant increase in tobramycin resistance was observed	Tobramycin was associated with significantly increased dyspnea, chest pain, and wheezing compared with the placebo group
Haworth et al^(72)^	Multicenter, randomized, controlled trial	Bronchiectasis patients with chronic *P. aeruginosa* infection (*n* = 144)	Nebulized colistimethate sodium 1,000,000 U BID or placebo (0.45% saline) BID for 6 months using an INeb device or until first exacerbation. Patients receiving oral macrolides at the start of the trial were continued on therapy	Time to first exacerbation, emergence of resistant organisms, *P. aeruginosa* CFUs	No significant difference between treatment and placebo in time to first exacerbation was observed (*p* = 0.11). Analysis of patients with ≥80% compliance showed that median time to first exacerbation was significantly increased; no emergence of resistant organisms. *P. aeruginosa* CFUs were reduced at weeks 4 and 12	Bronchoconstriction after the first dose in 7.5% of colistin patients versus 1.4% in patients receiving placebo. No significant difference in AEs (143 in 47 colistin patients versus 108 in 38 placebo patients, *p* = 0.25)

AE, adverse events; BID, twice a day; CFU, colony-forming units; DPI, dry powder for inhalation; EOT, end of treatment; NCFBE, non-cystic fibrosis bronchiectasis; PE, pulmonary exacerbation; QOL, quality of life; SD, standard deviation; TEAEs, treatment-emergent adverse events.

Several phase 2 studies have evaluated the use of inhaled formulations of ciprofloxacin. A phase 2 trial of ciprofloxacin DPI at a dose of 32.5 mg twice a day showed a significant 3.6 log reduction in total sputum bacterial load versus placebo (*p* < 0.001) at the end of treatment, but no significant difference in exacerbation rates (*p* = 0.605; Table 3).^(53)^ ORBIT-1 evaluated ciprofloxacin for inhalation (150 mg of ciprofloxacin in 3 mL or 100 mg ciprofloxacin in 2 mL), while ORBIT-2 investigated a dual-release formulation of ciprofloxacin combining liposomal ciprofloxacin for inhalation (150 mg in 3 mL) with free ciprofloxacin (60 mg in 3 mL). Both trials demonstrated potent antipseudomonal activity (4 log reduction in density of *P. aeruginosa*; Table 3) and, in ORBIT-2, there was an increased median time to first exacerbation (by 76 days) in the per-protocol group compared with placebo (*p* = 0.046).^(59,131)^ Safety results from ORBIT-2 showed a similar incidence of overall adverse events for subjects who received the dual-release ciprofloxacin formulation and placebo.^(131)^

Both the dual-release (ORBIT-3 and ORBIT-4) and DPI formulations of ciprofloxacin (RESPIRE-1 and RESPIRE-2) have been tested in phase 3 trials; the results were recently presented but not published at the time of submission of this article.

RESPIRE-1 included adults with a positive sputum culture for predefined bacteria and a history of treatment of at least two exacerbations in the previous 12 months. Patients (*N* = 416) randomly received inhaled ciprofloxacin DPI (32.5 mg) or placebo twice daily administered in either 12 cycles of 14 days on/14 days off, or in 6 cycles of 28 days on/28 days off, for 48 weeks. The investigators used a 2:1 active therapy versus placebo randomization schedule. Primary outcome measures were time to first exacerbation and frequency of exacerbation. Exacerbations were strictly predefined as the worsening of at least three respiratory symptoms (dyspnea, wheezing, cough, increased sputum volume over 24 hours, or increased sputum purulence) plus fever or malaise/fatigue, and systemic antibiotic use. Both primary endpoints were met with the 14-day on/off regimen. There was a 39% reduction in the number of exacerbations with the 14-day regimen over placebo (adjusted incidence rate ratio [IRR] 0.61; *p* = 0.0061); furthermore, active treatment significantly prolonged time to first exacerbation (adjusted hazard ratio [HR] 0.53; *p* = 0.0005), with a mean time to first exacerbation >336 days with active treatment versus 186 days in the two placebo arms (which were pooled for analysis). The 28-day regimen also reduced the number of exacerbations versus placebo, but the difference was not statistically significant (adjusted IRR 0.98, *p* = 0.89). Furthermore, there was a trend for a delay in the time to first exacerbation for those randomized to inhaled ciprofloxacin versus placebo on the 28-day on/off schedule (adjusted HR 0.73*; p* = 0.065). Serious treatment-emergent adverse events were similar with the 14-day regimen (16.9%), the 28-day regimen (19.9%), and placebo (23.4%).^(132)^

In RESPIRE-2, 521 patients were randomized to either ciprofloxacin DPI (32.5 mg) or placebo BID in a 2:1 ratio, using either a 14-day on/off or a 28-day on/off regimen over 48 weeks. Endpoints were evaluated using analyses with different alpha levels. The primary endpoints were time to first exacerbation versus pooled placebo and frequency of exacerbation versus matched placebo. Ciprofloxacin DPI did not significantly prolong time to first exacerbation versus pooled placebo using either of the two regimens (HR = 0.87, *p* = 0.40 and HR = 0.71, *p* = 0.05, respectively). A reduction in exacerbation frequency versus matched placebo did not reach predefined significance thresholds. Ciprofloxacin DPI was well tolerated in both regimens.^(133)^

A once-a-day inhaled formulation of liposome-encapsulated ciprofloxacin (150 mg/3 mL) and free ciprofloxacin (60 mg/3 mL, ARD-3150; Aradigm Corp., Hayward, CA) was recently evaluated in two double-blind, placebo-controlled phase 3 trials (ORBIT-3; *n* = 278 and ORBIT-4; *n* = 304) over 48 weeks in NCFBE patients chronically infected with *P. aeruginosa.* The trials consisted of six cycles of 28 days on and 28 days off treatment. The key efficacy endpoints were time to first pulmonary exacerbation (protocol defined) and the frequency of all and severe pulmonary exacerbations (defined as requiring treatment with IV antibiotics and/or hospitalization). Recently presented findings of pooled data showed that ARD-3150 increased median time to first exacerbation and reduced the frequency of protocol-defined pulmonary exacerbations and severe pulmonary exacerbations compared to placebo.^(134–136)^ In addition, ARD-3150 reduced sputum *P. aeruginosa* density without attenuation of ciprofloxacin activity and *P. aeruginosa* MIC, which remained stable throughout each on-treatment period. Rates of treatment-emergent adverse events and serious adverse events were similar across treatment arms in both studies.^(134–136)^

Some of the studies cited above^(2,53,59,131,134,136)^ provide evidence that sputum bacterial density was reduced with inhaled ciprofloxacin. A significant decrease in *P. aeruginosa* bacterial load was associated with trends for reduction in the risk and frequency of exacerbations.^(134,136)^ Serious adverse events were similar between active treatment and control groups.^(2,53,59,131,134–136)^ To achieve the maximum benefits from inhaled antibiotic therapy, further research could help to identify optimal drug regimens in specific groups of patients with NCFBE.

### Safety of inhaled antibiotics

A meta-analysis of eight trials involving 590 patients reported an acceptable safety profile for inhaled antibiotics (amikacin, aztreonam lysinate, ciprofloxacin, gentamicin, colistin, or tobramycin), with a withdrawal rate for inhaled therapy that was similar to that of the control group.^(99)^ The most frequent adverse event was bronchospasm, which occurred in 10% of patients receiving inhaled antibiotic therapy versus 2.3% in the control group (RR 2.96 [95% CI 1.30–6.73]; *p* = 0.01).^(99)^ Patients who received inhaled aminoglycosides were five times more likely to develop bronchospasm than those in the placebo group or those in the symptom-based therapy group (RR 4.78 [95% CI 1.55–14.76], *p* = 0.007). In contrast, the risk of bronchospasm was not significantly increased with inhaled ciprofloxacin (RR 1.07 [95% CI 0.25–4.56], *p* = 0.93) or colistin (RR 4.86 [95% CI 0.58–40.59], *p* = 0.14).^(96)^ Systemic absorption of inhaled antibiotics is variable but there is a potential for development of systemic side effects.^(137,138)^

### Limitations of inhaled antibiotic therapy in NCFBE

Most clinical trials investigating the use of inhaled antibiotic therapy in NCFBE only involved a small number of patients and were of short duration. Compliance with inhaled antibiotic therapy was not ideal in these studies, and there is currently no clear consensus on the endpoints to evaluate in clinical trials (microbiological vs. clinical efficacy). The full results of the recently completed phase 3 trials of ciprofloxacin in different formulations, DPI and liposome-encapsulated/dual-release, with a higher number of patients and of longer duration should advance our knowledge of choosing appropriate patient populations and different endpoints in the future. Overall, the benefits of inhaled antibiotics in NCFBE have been modest, but favorable trends should be further explored.

Oral/intravenous antibiotics in conjunction with inhaled antibiotics could lead to early eradication of *P. aeruginosa* in both CF and NCFBE patients, and this may reduce the frequency of exacerbations and improve quality of life.^(22,139)^ However, the value of adding an inhaled antibiotic for treatment of an acute exacerbation has not been established.^(95)^ In a retrospective study, 31 patients with NCFBE who were successfully treated with chronic inhaled antibiotics were compared with an age- and sex-matched cohort of 60 patients who were not treated.^(140)^ The study cohort had a greater number of exacerbations in the year before start of inhaled antibiotics, had a significantly lower lung function at baseline, and had higher scores on the BSI. However, in the year after initiation of inhaled antibiotics, the study group had significantly fewer exacerbations (*p* = 0.003) compared with the control cohort.^(140)^ The authors proposed using inhaled antibiotics in patients with NCFBE who suffered repeated exacerbations despite appropriate use of chronic oral macrolide therapy, aerosolized hypertonic saline, and airway clearance therapies. Selected patients with NCFBE may benefit from inhaled antibiotic therapy, and therapy may be more successful if targeted to specific patient groups.

## Conclusions

NCFBE is an underdiagnosed chronic inflammatory lung disease that is associated with significant morbidity and mortality, for which no licensed therapies are currently available. The most severe forms of NCFBE are associated with chronic *P. aeruginosa* infections and often require long-term antibiotic therapy that may increase the risk of antibiotic resistance. Inhaled antibiotics could have a role in treatment of initial infection with *P. aeruginosa* with the aim of eradicating the infection. Chronic infection with *P. aeruginosa* is much more difficult to eradicate, and the goal of antibiotic therapy is to reduce the bacterial load, reduce airway inflammation, and prevent exacerbations. Long-term therapy with oral macrolides is effective in this respect and supplementation with inhaled antibiotics could augment their efficacy in patients with severe illness or in patients infected with multidrug-resistant organisms. Evaluation of the liposomal dual-release formulation of ciprofloxacin in phase 3 clinical trials reduces the *P. aeruginosa* bacterial load, with a trend toward reduced risk of exacerbations. Inhaled antibiotics have antipseudomonal activity, have fewer systemic side effects, and they could provide alternative treatment options to address some of the treatment challenges that exist in the management of severe cases of NCFBE.

## Note Added After Online Publication

Since online publication of the final accepted version of this article, there have been a couple of important updates regarding the evidence for the use of inhaled antibiotics as therapy for bronchiectasis. First, in September 2017, the European Respiratory Society (ERS) published guidelines for the management of adult bronchiectasis.^(145)^ Despite no approved inhaled antibiotic for noncystic fibrosis bronchiectasis (NCFBE) being available, they suggest offering long-term inhaled antibiotic treatment for adults with bronchiectasis and chronic *Pseudomonas aeruginosa* infection with three or more exacerbations per year. They consider it a conditional recommendation based on moderate-quality evidence. In addition, they suggest long-term treatment with an inhaled antibiotic for patients not infected with *P. aeruginosa* infection with three or more exacerbations per year that do not respond to or tolerate oral antibiotic prophylaxis (conditional recommendation, low quality of evidence).

The ERS recommendations also include the use of antibiotics, including inhaled formulations, as an option for eradication treatment in patients with a first/new isolation of *P. aeruginosa*; however, this is based on very low-quality evidence. Nonetheless, this review is timely in light of these recommendations.

Second, in this review of various studies evaluating use of inhaled antibiotics in NCFBE, the promise of large clinical trial programs for inhaled ciprofloxacin has been alluded to, including the RESPIRE trials (now published in full^(146,147)^) with twice-daily ciprofloxacin DPI, and the ORBIT trials with nebulized once-daily dual-release liposomal ciprofloxacin.^(148,149)^ There was much anticipation that one or both of these products would result in the first Food and Drug Administration (FDA)-approved inhaled antibiotic to offer to patients with advanced NCFBE. However, this does not appear to be imminent, as the FDA's Antimicrobial Drugs Advisory Committee recently voted against a recommendation for approval of both products.^(150,151)^

In a press release on January 29, 2018, Aradigm Corporation announced that the FDA did not approve ARD-3150 “in its present form” and provided recommendations for resubmission of the application. Bayer has not disclosed any details of the FDA's complete response letter pertaining to the new drug application for ciprofloxacin DPI. Both companies are expected to submit applications for their inhaled antibiotic formulations to the European Medicines Agency (EMA) for approval in Europe.

These recent developments highlight key challenges for this therapeutic field, including the importance of establishing consensus regarding the most meaningful clinical endpoint for NCFBE in future trial designs. For the FDA submissions, the primary endpoint in both the RESPIRE^(146,147)^ and the ORBIT^(148,149)^ trials was time to first pulmonary exacerbation over 48 weeks. This endpoint has limitations, since it is unclear whether a treatment-mediated delay in time to first exacerbation over 48 weeks may be clinically meaningful to patients who are likely to be on this treatment for a considerable length of time. Reducing exacerbation frequency (the primary endpoint for any potential EMA application) may be more clinically meaningful for long-term treatment. In this context, it is worth noting that a meta-analysis of pooled data from each RESPIRE-1/RESIRE-2^(152)^ and from ORBIT-3/ORBIT-4^(148)^ evaluating frequency of exacerbations significantly favored the respective ciprofloxacin formulation over placebo.

Given the aforementioned pooled trial data and the favorable safety/tolerability profile of inhaled ciprofloxacin formulations, inhaled antibiotics are of benefit in some patient groups. More stringent inclusion criteria for future trials based on more clearly defined subgroups of patients and better methods of distinguishing these subgroups may be the way forward in the fight to find a licensed treatment for this debilitating orphan disease. At present, the lack of an approved therapy limits implementation of the ERS recommendations, and clinicians are awaiting clearance of one or more inhaled antibiotics as an additional therapeutic option for selected patients with NCFBE.
